# Microstructure and Mechanical Properties of AlCoCrFeNi High-Entropy Alloy-Reinforced Ti-6Al-4V Composites

**DOI:** 10.3390/ma18133179

**Published:** 2025-07-04

**Authors:** Abdulaziz Kurdi, Animesh Kumar Basak, Nachimuthu Radhika, Ahmed Degnah

**Affiliations:** 1 Advanced Materials Technology Institute, King Abdulaziz City for Science and Technology, P.O. Box 6086, Riyadh 11442, Saudi Arabia; akurdi@kacst.gov.sa; 2 King Salman Center for Disability Research, Riyadh 11614, Saudi Arabia; 3 Adelaide Microscopy, The University of Adelaide, Adelaide, SA 5005, Australia; 4 Department of Mechanical Engineering, Amrita School of Engineering, Amrita Vishwa Vidyapeetham, Coimbatore 641112, India; n_radhika1@cb.amrita.edu

**Keywords:** high entropy alloy (HEA), metal matrix composite (MMC), microwave sintering, microstructure, micro-pillar compression, disability

## Abstract

High-entropy alloy (HEA) particle-reinforced metal matrix composites (MMCs) are a new generation of MMCs with potential applications as orthopedic material in automotive, aerospace, and biomedical fields. In this study, AlCoCrFeNi HEA-reinforced Ti-6Al-4V metal matrix composites (MMCs) were prepared by microwave sintering. The microstructural aspects of the MMC were investigated by scanning electron microscopy (SEM) and transmission electron microscopy (TEM), with an emphasis on the interdiffusion (ID) layer. The mechanical properties of the composites were studied by micro-pillar compression at the micro-length scale. The results show that the ID layer exists between the HEA particles and the matrix, is equiaxed in nature, and leads towards metallurgical bonding within the composite. The strength of this ID layer (1573 MPa of yield strength and 1867 MPa of compressive strength) and its Young’s modulus (570 MPa) were about 1.5 times lower than that of the matrix. The HEA particles exhibit the highest strength (2157 MPa of yield strength and 3356 MPa of compressive strength) and Young’s modulus (643 MPa), whereas the matrix falls in between 2372 MPa of yield strength and 2661 MPa of compressive strength, and a Young’s modulus of 721 MPa.

## 1. Introduction

Metal matrix composites (MMCs) are one of the most prominent engineering materials where the matrix metal/alloy is reinforced by incorporating various second-phase particles/fibers. MMCs are well recognized for high hardness, modulus, and specific strength [1,2] compared to their respective constituent phases. Among a wide range of metallic materials, Ti-6Al-4V is well known for its diverse applications, which require structural strength [3], corrosion resistance [4], and high-temperature oxidation resistance [5,6,7]. They also account for a large quantity of aerospace and biomedical-related applications [8]. The inherent higher specific strength and stiffness of this alloy could be exploited further by incorporating particles as reinforcing elements [9,10]. The usual reinforcements are different types of ceramic particles, for example, SiC, Si_3_N_4_, TiB, TiC, WC [11,12], and others, known for their high hardness. However, the main challenges are the development of a weak particle/matrix interface and significant differences in the thermal expansion coefficient among the matrix and reinforcement phases [13]. One of the ways to tackle this challenge is to introduce a natural metal–metal interface in the MMCs by incorporating metallic particles, such as high-entropy alloy (HEA) particles [14,15,16,17]. High-entropy alloys (HEAs) are different from traditional multi-component alloys, as the former contain at least five major elements of ‘equiatomic’ amounts with simple solid solution structures [18]. HEAs possess superior strength, together with enhanced wear resistance, thermal stability [19,20], and higher elongation, than that of ceramics and metallic glasses [17,21,22]. These unique characteristics make HEAs potential reinforcement candidates towards the development of unique HEA particles that reinforce MMCs. In addition, the coefficient of thermal expansion of the HEA and metal matrix falls in a close range [23,24]. The CoCrFeNi-X series of HEA systems is one of the most widely studied, as reported in the literature, where X represents one of the following elements according to the intended applications: Mn, Mo, Al, Cu, or Ti. These new generations of MMCs have potential applications in the automotive and aerospace industries [25]. In addition, as an established application of metallic biomaterials for implants (e.g., fixing plates, rods, screws, etc.), such MMCs can also be used in biomedical applications related to musculoskeletal disability treatments [25,26]. Earlier studies involved the development of aluminum (Al) matrix composites by incorporating a number of HEAs, such as CoCrFeNi [27], Al0.8CoCrFeNi [28], AlCoCrFeNi [29], CoCrFeMnNi [30], and FeCoNi1.5CrCu [31]. These studies exhibit different elemental distributions/formations at the particle/matrix interface. The interface usually contains intermetallic compounds rather than widely speculated solid solutions, which constrain the deformability of the alloy [32]. Wang et al. [33] proposed FeNiCrCoAl HEA-reinforced aluminum (Al 2024) matrix composites, with a compressive strength of 710 MPa, much higher than the matrix material alone. Chen et al. [14] chose an AlCoNiCrFe particle-reinforced copper (Cu) matrix and reported a huge 160% increase in yield strength with 15% elongation. Liu et al. [29] reported the ‘transition layer’ formation at the interface of AlCoCrFeNi HEA-reinforced aluminum composites and confirmed the FCC structure of the ‘transition layer’ [29]. Satyanarayana et al. [34] studied the effect of heat treatment temperature on Al0.5Si0.5FeCoNi HEA particle-reinforced titanium (Ti) matrix composites. Yuan et al. [30] and others [35] studied CoCrFeNiMo0.2 HEA particle-reinforced titanium (Ti) matrix composites and subjected them to different sintering temperatures. This sintering temperature showed an effect on the formation and thickness of the interface layer.

Irrespective of the matrix, reinforcement phases, and material system, it was reported that a diffusion layer formed at the matrix/reinforcement interfaces, commonly known as the ‘interdiffusion (ID) layer’ in the literature [30]. This ID layer holds the key towards cohesion within the composite system and dictates the overall mechanical properties of the composite in general. This layer is metallurgical in nature, and these bonding aspects of this layer among the matrix and reinforcement phases have an obvious role in the mechanical properties of the composite structure [24,36]. The only mechanical property reported on this ID layer was the hardness value via nanoindentation. Keeping this in mind, the stress–strain behavior of this ID layer was investigated in this study. The specific knowledge gap addressed was the mechanical properties of this ID layer beyond hardness evolution. Most of the work reported in the literature is on HEA particle-reinforced pure metals as a matrix [14,30,36], with few reports on Ti-6Al-4V as a matrix material [32,37]. In previous communications, a comprehensive overview of the micro-scale mechanical properties of AlCoCrFeNi HEA [38] and the tribological behavior of microwave-sintered AlCoCrFeNi HEA-reinforced Ti-6Al-4V MMC [37], together with microstructural characterization, have been reported. However, detailed characterization and the ID layer’s role in the interfacial bonding characteristics and strengthening/toughening mechanisms between the HEA particles and the matrix have not been addressed.

In view of this, the aim of the present study was to investigate the microstructural and micro-mechanical aspects of the interdiffusion layer (ID) further, which is the novelty of the current work. In addition to this, a first attempt was taken to characterize the strength of the interdiffusion layer, via micro-pillar compression [39,40]. This was otherwise not achievable, as the thin interdiffusion layer profile does not permit preparation of the traditional ‘dog-bone’-shaped tensile specimens. Together with the microstructural characterizations, the micro-mechanical properties of the composite were explored in addition to exploring the associated deformation mechanisms.

## 2. Experimental

### 2.1. HEA Particle Reinforced MMC Fabrication

Commercially available Ti-6Al-4V powder, with a particle size of 20–50 μm and irregular morphology, was used as the matrix material [37]. Equiatomic AlCoCrFeNi HEA was synthesized by using a gas atomization process and the outcome was spherically-shaped particles, 20–25 μm in diameter [38,41]. The mass ratio of the HEA and Ti-6Al-4V powder was 8:92, as optimized to attain higher mechanical properties, as reported in previous communications [37]. Both powders were mixed through low-energy ball milling for 1 h to obtain a homogenous composition. The ball-to-mass ratio was 10:1, and the speed was 200 rpm. To prevent strong agglomeration of the powder, ball milling was conducted in a vacuum environment. The resulting composite mixture was compacted to produce a solid mass in the form of a cylinder (10 mm in diameter and 10 mm in height), achieved by applying a pressure of 950 MPa, with the help of a hydraulic press. The resulting mass was sintered by microwave sintering for 1 h at 850 °C, with the help of 2.45 GHz 5 kW microwave equipment [38]. The sample was placed inside a high-purity quartz (glass) tube to ensure microwave transparency, and to minimize external contamination. The heating rate was maintained at approximately 20 °C/min, performed under a continuous Ar atmosphere to prevent oxidation. A detailed characterization of the powders and the composites, including phase analysis, is reported in a previous communication [37]. Compared to conventional hot-pressing or spark plasma sintering, microwave sintering offers distinct advantages in processing advanced materials. The sintering enables uniform heating, reduced thermal gradients, lower energy consumption, and rapid sintering, which significantly contributes to finer grain structures and faster densification, due to suppressed grain growth. Additionally, the use of an inert Ar atmosphere during microwave sintering minimizes contamination. These attributes make microwave sintering particularly suitable for fabricating high-performance materials, such as HEA-reinforced composites [42,43].

### 2.2. Specimen Preparation for Microscopy

The preparation procedure of the sintered specimen for electron microscopy was as follows: “The as-fabricated cylindrical shaped specimens were cut in half in the middle and mounted in a resin block by a hot-mounting process (Cito press-10, Struers, Denmark). Then, the blocks were grinded and polished in polishing cloths, with varying polishing slurries, in the Struers automatic metallographic polisher. The final polishing was conducted in a colloidal silica to achieve scratch-free polished surfaces. The microstructural characterization was conducted with a field emission scanning electron microscope (FESEM, Quanta 450 FEG, Thermo-fisher scientific, USA), in both the secondary electron (SE) and backscattered electron (BSE) modes. The elemental analysis was conducted by energy dispersive spectroscopy (EDS) with the help of the Oxford EDS system (Oxford Instruments, UK), attached with the SEM. The TEM foils were prepared by using a focused ion beam (FIB)-SEM (Helios Nano-lab 600, Thermo-fisher scientific, USA), via an in situ lift-out technique, and the TEM investigation was conducted using a probe-corrected transmission electron microscope (TEM), Titan Themis (Thermo-fisher scientific, USA), operated at 200 kV” [38].

### 2.3. Micro-Pillar Fabrication and Compression in SEM

Several micro-pillars were prepared by FIB-SEM on three different regions: (i) on the ID layer, (ii) on the HEA particles, and (iii) on the Ti-6Al-4V matrix. The dimensions of the micro-pillars were 9 μm in length and 3 μm in diameter, with a slightly tapered appearance, which was less than 2°, due to the material–ion beam interactions [44,45]. The details on the FIB-SEM procedure of micro-pillar fabrication can be found in previous communications [46,47].

The in situ nanoindentation system, which was used to compress the micro-pillars, was a PI 88 from Hysitron Inc. (Eden Prairie, MN, USA), where the nanoindentation tip was replaced by a 5 µm diameter flat punch. The loading and unloading rates were 3 and 50 nm/s, respectively, and the whole compression process was recorded via video with secondary electron images. The applied load (*F*) and resulted changes in pillar height (Δ*l*) were recorded in real-time by the Hysitron software (V 10.2.1). Later on, these load–displacement curves were translated into stress–strain curves, according to the method proposed in the literature, by incorporating all of the related corrections [48,49]. Multiple micro-pillars were fabricated and compressed for a given specimen (at least five) to ensure data reproducibility. In order to calculate the stress–strain curves, the following procedure was employed: the applied normal force (*F*) and corresponding changes in the pillar length (Δ*l*) were recorded during compression by a computer-controlled program and subsequently used to calculate the engineering stress and strain, according to Equations (1) and (2):

(1) $$\sigma =\frac{F}{A_{0}}$$

where σ is the engineering stress, *F* is the normal force, and *A*_0_ is the cross-sectional area of the pillar at 25% of its height from the top. As the pillars were slightly tapered (<2°), the most likely deformation will therefore happen closer to the top surface [39].

(2) $$\varepsilon_{E}=\frac{∆l}{l_{0}}$$

where *ε_E_* is engineering strain, Δ*l* is change in pillar length, and *l*_0_ is initial pillar length. Details of the equations can be found in the literature [48,50]”.

## 3. Results and Discussion

### 3.1. Microstructural Investigation by Scanning Electron Microscopy (SEM)

**Figure 1:**
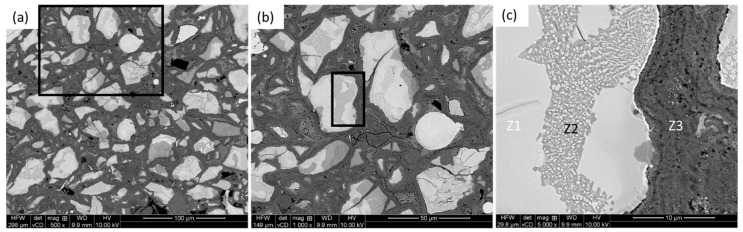
Back-scattered electron (BSE) images of the composite: (**a**) overall view, and (**b**) magnified view of the location marked in (**a**), further detailing the marked region in (**c**) exhibiting different zones: HEA particle (Z1), ID layer (Z2), and matrix (Z3).

Figure 1 shows the microstructure of the AlCoCrFeNi HEA particle-reinforced Ti-6Al-4V MMC, after metallography polishing at different magnifications, in back-scattered electron (BSE) imaging mode. The BSE imaging mode enhanced the contrast among the reinforcement particles (bright) and matrix (gray) due to atomic number difference in the associated elements. Figure 1a,b confirms the homogeneous distribution of the reinforming particles, which are irregular in shape, in the matrix. A further look at one of the HEA particles (Figure 1c) shows the trail of distinct ID layers and different zones, which were labeled as follows: zone 1 (Z1) was the HEA particle itself, zone 2 (Z2) was the ID layer, and zone 3 (Z3) was the matrix.

As evident from Figure 1c, the ID layer (Z2) zones were well bonded with the matrix and extend at various depths towards the HEA particles. The reason behind this was the ball milling of the powders, which offered a good homogeneous blend of the powder mixture. Thus, there was a significant extent of diffusion, as opposed to ‘limited diffusion’, as claimed by Yuan et al. [30], Xiong [32] and Qiang et al. [36]. There were two different aspects observed: (i) powder consolidation process and (ii) retention of the original shape of the reinforcing phases. In the present case, the microwave sintering technique was used after compaction, unlike spark plasm sintering [30,32,36]. Secondly, though the original shape of the HEA reinforcing particles was spherical after gas atomization, they became irregular in shape after ball milling through fragmentation.

**Figure 2:**
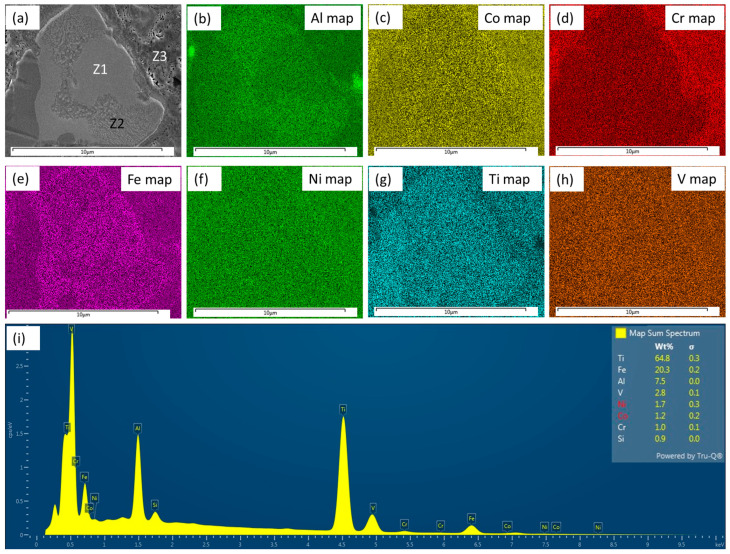
(**a**) Secondary electron (SE) image corresponds to the location of the elemental mapping of different elements; (**b**–**i**) overall elemental analysis.

As also evident from Figure 1c, the ID layers did not form around all the HEA materials to the same extent, confirming the uneven and preferential diffusion (perhaps channeling) of the elements. The elemental distributions of the HEA particle, partial/matrix interface, and the matrix were further investigated by elemental mapping, as depicted in Figure 2, together with overall elemental analysis.

The three elements Co, Ni, and V diffused to a certain extent in the HEA particles, and probably formed the solid solution compound (known as FCC stabilizers [51]), as investigated further by TEM and reported in Section 3.2. Comparing the element distribution results, it seems that the diffusion took place to a higher extent, as mentioned earlier, and that the distinct appearance of the ID layer was not visible as reported by other researchers [30,32,36]. In addition to this, there was no obvious delamination, cracks, or defects between the interface layers, which confirmed the better wetting and cohesion amongst the particles and matrix. Details of the power characterization are reported in our previous communication [37] and thus avoided here.

### 3.2. Microstructural Investigation by Transmission Electron Microscopy (TEM)

**Figure 3:**
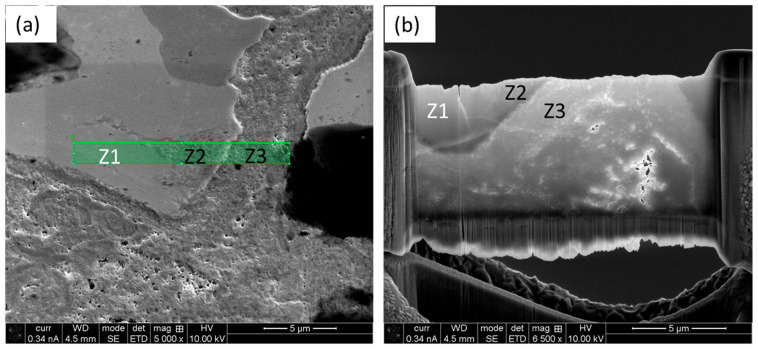
(**a**) Representative SE image with the overlay of the TEM foil area, as marked with the green box, and (**b**) TEM foil exhibiting the presence of different zones (Z1, Z2, and Z3) as described previously.

The location of the TEM foil was selected in such a way that it comprised a portion of HEA particle (Z1), ID layer (Z2) and matrix (Z3), as shown by the ‘green box’ in Figure 3a, showing representative microscopic views of the composite. The prepared TEM foil is shown in Figure 3b, which clearly identifies the different zones, as stated earlier.

**Figure 4:**
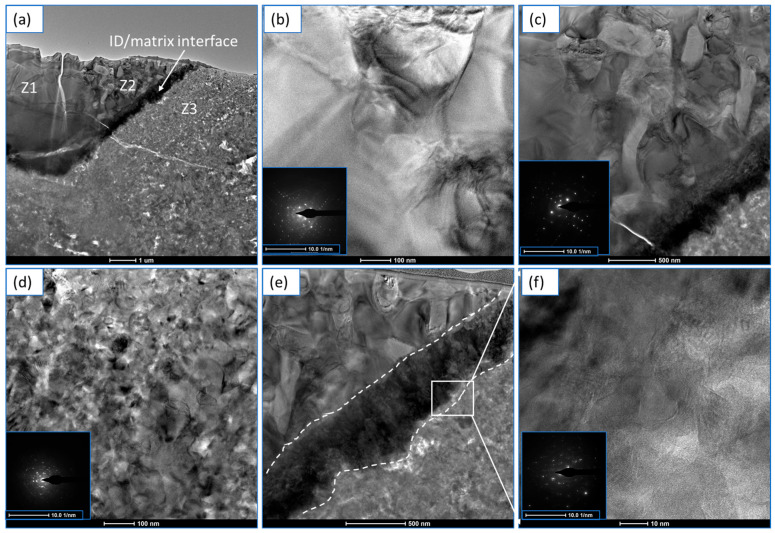
Representative TEM images of the composite: (**a**) overall morphology of the composite with the presence of different zones (Z1, Z2 and Z3) as outlined previously; (**b**) morphology of Z1; (**c**) morphology of Z2; (**d**) morphology of Z3; (**e**) morphology of ID/matrix interface as confined within white dotted lines; and (**f**) HR-TEM image of the ID/matrix interface as marked in (**e**). The representative SEAD patterns are shown as inserts.

The outcome of the TEM investigation is depicted in Figure 4, and the presence of the different zones is annotated accordingly (Figure 4a). Clearly, there was a distinct interface between the matrix and ID layer (as marked in Figure 4a). The morphology of the HEA particle (Figure 4b) showed a typical dendritic structure [52].

The dendritic structure in the ID layer was gradually transformed into equiaxed crystals (Figure 4c) with no apparent orientation. The dendrite growth process was limited due to the constraint of the surroundings, and they tended to grow into equiaxed crystals. The matrix (Figure 4d) showed the random distributions of different crystals of the constituent elements. The ID/matrix interface (Figure 4e) clearly demonstrates the formation of columnar grains, which eventually transformed into equiaxed grains as the interface progressed through the diffusion process. The high-resolution (HR) TEM image of the interface (Figure 4f) shows the coherency of the structure at the atomic scale and confirms solid-state diffusion [53]. For the first time, the existence of such columnar grains on the ID/matrix interface are reported in this work. In addition to solid-state diffusion, the role of thermal expansion mismatch between HEA (12.7 × 10^−6^/°C) and Ti-6Al-4V (8.6 × 10^−6^/°C) could also influence the formation of the ID layer, which requires further investigation.

The TEM images were accompanied by respective selected areas of electron diffraction (SAED) patterns (as inserts), and they confirmed the crystalline nature of the structure, irrespective of different zones. Moreover, it was interesting to note that both the ID layer and the ID/matrix interface contained an orderly FCC structure over the formation of intermetallic compounds. Thus, the formation of the ID layer at the combined ID/matrix interface was due to the exposure to local high temperatures during sintering.

### 3.3. Mechanical Properties Investigation by Micro-Pillar Compression

**Figure 5:**
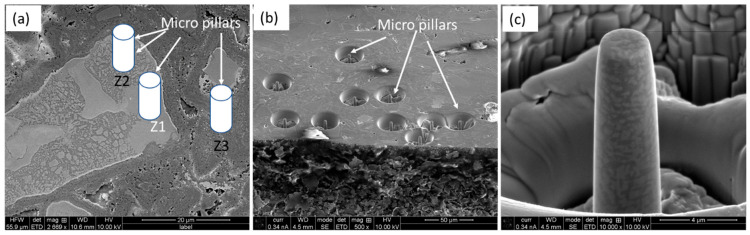
(**a**) Representative SE image of the composite with overlayed position of the micro-pillars in different zones (Z1, Z2, and Z3); (**b**) FIB-SEM prepared micro-pillars in different regions of the composites; and (**c**) high-magnification image of a representative micro-pillar in the ID layer (Z3).

The micro-pillars were fabricated carefully to represent the different zones, namely, Z1, Z2 and Z3, as mentioned earlier. Figure 5a shows a representative SE image of the composite with the positions of the micro-pillars at different zones overlayed. The physical appearance of the as-fabricated FIB-SEM micro-pillars in different regions of the composites is shown in Figure 5b, and a representative high-magnification image of the micro-pillars at the ID layer (Z2) is depicted in Figure 5c, where the equiaxed grain arrangements are evident.

**Figure 6:**
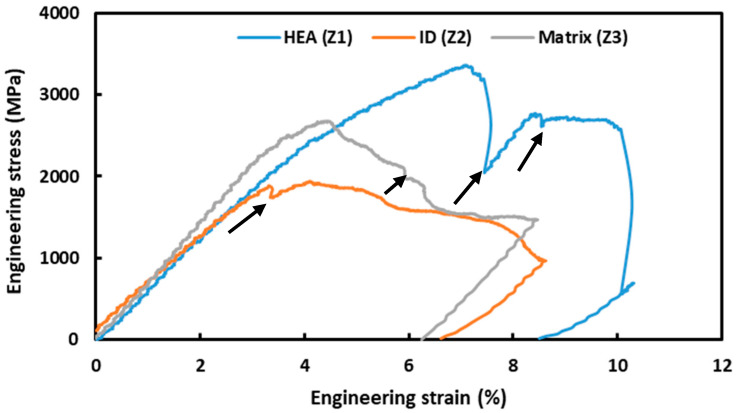
Stress–strain curves at different zones of the composite: HEA particle (Z1), interdiffusion (ID) layer (Z2), and the matrix (Z3). The stress drop occurrences are pointed out by black arrows.

During in situ micro-pillar compression, the corresponding load–displacement evolution was recorded by a data acquisition software and later converted into stress–strain curves, as depicted in Figure 6. Though several micro-pillars were compressed and analyzed in the zones, only one representative curve for each given zone is reported in Figure 6, for neatness and ease of comparison. As can be seen from Figure 6, at the start of loading, the stress level increases linearly to that of the strain, until the material reaches its yield point. Beyond this point, there were several stress drops, which correspond to the formation and propagation of the slip planes, as observed in the ‘live view’ of the compression process. This process continues until complete fracture of the micro-pillars. It is obvious from Figure 6 that the HEA particles showed the highest strength, followed by the matrix and the ID layer. The micro-mechanical properties, such as the yield strength (YS), ultimate compressive strength (UCS), and Young’s modulus, were calculated from the stress–strain graphs and are tabulated in Table 1, together with the standard deviations.

As can be seen from Figure 6 and Table 1, the matrix exhibits the highest strength and elastic modulus, followed by the HEA particle. The ID layer showed the lowest strength (1573 MPa of YS and 1867 MPa of UCS), as well as elastic modulus (570 MPa), which was about 1.5 times lower to that of the matrix. This trend was not unexpected, as Ti-6Al-4V is one of the high-strength materials [54]. It was not possible to make a one-to-one comparison of these data to those in the literature, as this is the first report of its kind. Having said that, the general hardness evolution of this ID layer on other material system, as reported in the literature, is stated hereafter.

Yuan et al. [17] reported that when the sintering temperature was 850 °C, the hardness was 402.6 HV, the yield strength was 928.2 MPa, and the compressive strength was 2032.6 MPa for AlCoCrFeNi HEA particle-reinforced titanium subjected to macro-scale compression tests. “With the increase of the sintering temperature, the high entropy alloy particles had a greater degree of dissolution and diffusion, which reduced the strengthening effect of the particles and the mechanical properties” [30]. Yuan et al. [17] investigated CoCrFeNiMo_0.2_ HEA particles (20–45 µm) as the reinforcement and pure titanium powder (100 µm) as the matrix, by hot pressing sintering in vacuum. The hardness of the CoCrFeNiMo_0.2_ particles, diffusion layer, and matrix were in the ranges of 5–6 GPa, 11–13 GPa, and 4.5–5.5 GPa, respectively. Similarly, Xiong et al. [32] investigated the hardness of different zones that formed in a CoCrFeNiMo HEA-reinforced titanium matrix composite via spark plasma sintering at 1000 °C. They reported Vickers hardness values of 413, 379, and 388 HV, respectively, for the HEA particles, ID layer, and matrix. These followed the same trend, as found in the present case, where the HEA particles exhibited higher strength, followed by the ID layer and matrix.

### 3.4. Deformation of Micro-Pillars During Compression

**Figure 7:**
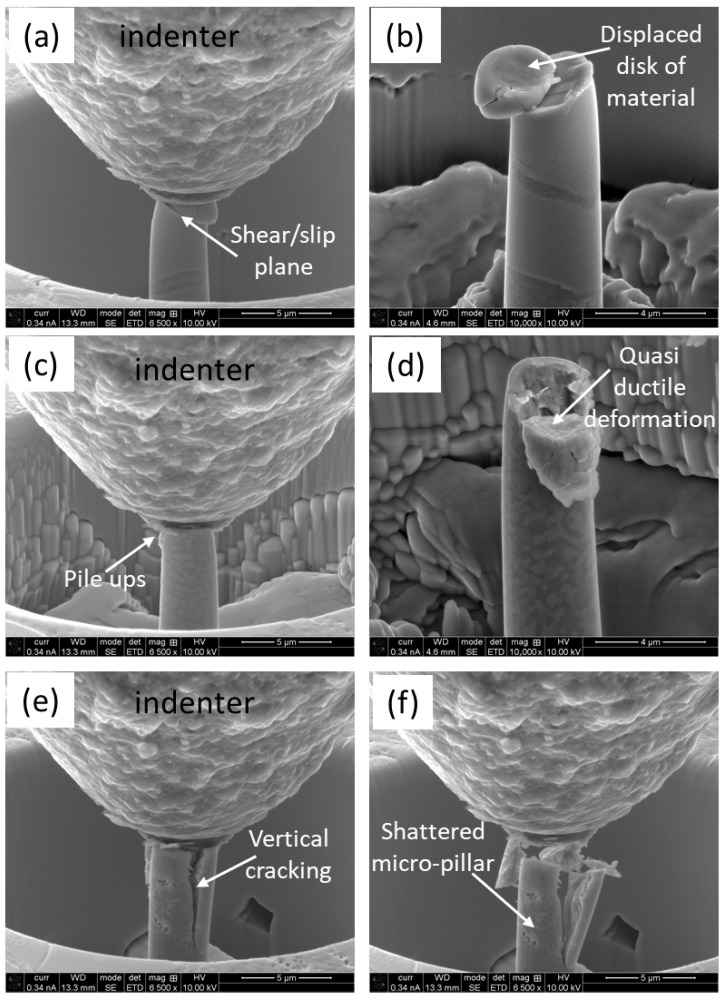
High-magnification SEM micrographs of the micro-pillars at 25% loading interval (left column) and upon completion of loading (right column): (**a**,**b**) on HEA particles, (**c**,**d**) on ID layer, and (**e**,**f**) on matrix.

As the loading continues during the compression test, the micro-pillars experience plastic deformation once the threshold of the yield point is reached. SEM micrographs were taken at different intervals of deformation, as well as after the completion of loading, to elucidate the prevailing deformation aspect. Figure 7 depicts a series of high-magnification SEM micrographs of a micro-pillar, at different zones, at 25% intervals, and after the completion of compression. In the case of the HEA particles (Figure 7a,b), the formation and propagation of the slip plane is evident, which caused a ‘disk’-type displacement of the physical material and resembled the representative deformation of this material under compression [55]. The scenarios were different for the ID layer, as shown in Figure 7c,d. In this case, the deformation comprised fragmentation and pulverization of the material, where the highest stress concentration took place. The stress was released in the form of plastic shear bands and networks of crack formation, which led to the eventual failure of the micro-pillar. In case of the matrix, the deformation was more expanded and forceful, as shown in Figure 7e,f. In this case, extended cracking took place, even under the 25% loading interval, which became more evident upon completing the unloading. Both interparticle and intraparticle collapses were evidenced, together with severe cracking, pile-ups, and localized quasi-brittle microfractures

## 4. Deformation and Strengthen Mechanism

Combined with research from related scholars on the same type of HEA particles, the reinforced metal matrix composites [56,57,58,59,60] deformation and reinforcement mechanisms were reflected in the following aspects: (1) The presence of different zones within the composite favors grain refinement (equiaxed grains) to accommodate plastic deformation. (2) As the HEA particles have higher stiffness than the matrix [25], there were effective load transfer mechanisms, with enhanced load-bearing capacities of the matrix. (3) The presence of hard reinforcing particles hinder dislocation movements, thereby improving the deformation resistance of the composites thanks to the Orowan strengthening [61]. (4) During the powder complication process (sintering), solid-state diffusion took place, resulting in the pinning effect, which hindered dislocation thanks to the solid solution strengthening [26]. (5) The formation of ID layers, together with the ID/matrix interface, metallurgical bonding between the particles, and the matrix prevail, significantly improved the mechanical properties of the composites. In addition to that, the presence of equiaxed grain structures in the ID layer improved toughness compared to columnar grain interfaces in traditional MMCs [61].

Moreover, the presence of an orderly FFC structure (Figure 4) was due to the high mixing entropy, which can significantly reduce free energy [21]. This favors solid solution formation over intermetallic compounds, as per the solidification process of multi-principal alloys. “The reason was that Ti, with a larger atomic radius, occupies the lattice position, the effect of solid solution strengthening increases significantly, with the increase of lattice distortion energy” [62,63,64]. In addition, “due to the mismatch of thermal expansion coefficient between HEA and the Ti-6Al-4V matrix [17], dislocations with high density and complexity occur in the matrix”. When the composite material was deformed, HEA particles prevented dislocation movement to enhance the material’s resistance to deformation, which ultimately resulted in increased strength. The lattice distortion probability of the base alloy increased with the increase in HEA particles. The dislocation of slip deformations is hindered by lattice distortion to a certain extent, and the strength of the material increases. All of these factors led to the microcracked, fragmented, and pulverized materials under compression stress due to intergranular and trans-granular fractures.

This study reveals the distinct microstructural responses of different zones of the composites to micro-pillar compression, with respect to deformation and damage, mechanical properties, and fracture geometries. In situ SEM observations of the compression processes of the different zones featured plastic deformation at the applied loading conditions. This feature was also confirmed by the continuous force–displacement curve, with regular stress drops beyond yield point, indicating the occurrence of shear band movement and cracking during compression. Even though no distinct discontinuities are evident in Figure 6, at a microstructural level, the plastic deformation in the different zones reflects entirely upon the different deformation characteristics. As a potential biomedical application, the in vitro cytotoxicity or corrosion behavior of these materials will be reported in future communications.

## 5. Conclusions

The formation of different zones in AlCoCrFeNi HEA-reinforced Ti-6Al-4V composites was investigated in this study, in the terms of physical and micro-mechanical characterizations. Emphasis was given to the interdiffusion (ID) zone characterization, as it holds the key towards a better cohesion of the reinforcing particles in the matrix and dictates the overall mechanical properties of the composites. Based on the experimental evidence, together with critical analysis of the data in view of the literature, the following conclusions can be drawn:

(1) The composite consists of a matrix, an interdiffusion (ID) layer, and HEA particles, which promote multiple strengthening mechanisms upon compression. The AlCoCrFeNi HEA particles in the composite experienced diffusion, which led to the formation of the interdiffusion (ID) layer, comprising equiaxed grains. ID layer/matrix interfaces also existed within the columnar grains, which transformed into equiaxed grains as the progression of the interface took place.

(2) The ID layer exhibited a lower strength (1573 MPa of YS and 1867 MPa of UCS) and Young’s modulus (570 MPa), which were about 1.5 times lower than that of the matrix. The HEA particles exhibited the highest strength (2157 MPa of YS and 3356 MPa of UCS) and Young’s modulus (643 MPa), whereas the matrix fell in between in terms of strength (2372 MPa of YS and 2661 MPa of UCS) and Young’s modulus (721 MPa).

(3) The deformation aspect of the HEA particles was quasi-ductile, due to their high stiffness, as evident by the formation and propagation of shear/slip bands. The ID layer suffered from micro-cracking and pulverization, whereas the matrix exhibited extended cracking under compression.

## Figures and Tables

**Table 1:** Mechanical properties of AlCoCrFeNi HEA particle-incorporated Ti-6Al-4V MMC at different zones, as outlined in Figure 1c.

Different Zones in the Composite	Yield Strength (σ_y_), MPa	Ultimate Compressive Strength (σ_UTS_), MPa	Elastic Modulus (E), MPa
HEA particle (Z1)	2157 ± 107	3356 ± 126	643 ± 36
Interdiffusion (ID) layer (Z2)	1573 ± 98	1867 ± 103	570 ± 34
Matrix (Z3)	2372 ± 118	2661 ± 172	721 ± 42

## Data Availability

The original contributions presented in the study are included in the article, further inquiries can be directed to the corresponding author.
